# Plasticity and Co-Factor-Dependent Structural Changes in the RecA Nucleoprotein Filament Studied by Small-Angle X-Ray Scattering (SAXS) Measurements and Molecular Modeling

**DOI:** 10.3390/molecules30081793

**Published:** 2025-04-16

**Authors:** Satomi Inaba-Inoue, Afra Sabei, Anne-Elisabeth Molza, Mara Prentiss, Tsutomu Mikawa, Hiroshi Sekiguchi, Chantal Prévost, Masayuki Takahashi

**Affiliations:** 1 Faculty of Advanced Life Science, Hokkaido University, Sapporo 060-0810, Japan; inaba@sci.hokudai.ac.jp; 2 Université Paris-Cité, CNRS UPR9080, Laboratoire de Biochimie Théorique, Institut de Biologie Physico-Chimique, F-75005 Paris, France; afra.sabei@ibpc.fr; 3 UMR URCA/CNRS 7369, Matrice Extracellulaire et Dynamique Cellulaire (MEDyC), Université de Reims Champagne-Ardenne, F-51100 Reims, France; 4 Department of Physics, Harvard University, Cambridge, MA 02138, USA; 5 RIKEN Center for Biosystems Dynamics Research, Yokohama 230-0045, Japan; 6 Japan Synchrotron Radiation Research Institute, Sayo-cho, Sayo-gun, Hyogo 679-5198, Japan; sekiguchi@spring8.or.jp; 7 School of Life Science and Technology, Tokyo Institute of Technology, Tokyo 152-8550, Japan; takahashi.m@kpu.ac.jp

**Keywords:** RecA nucleofilament, SAXS, helical protein assembly, protein filament plasticity, integrative modeling

## Abstract

Structural analyses of protein filaments formed by self-assembly, such as actin, tubulin, or recombinase filaments, have suffered for decades from technical issues due to difficulties in crystallization, their large size, or the dynamic behavior inherent to their cellular function. The advent of cryo-electron microscopy has finally enabled us to obtain structures at different stages of the existence of these filaments. However, these structures correspond to frozen states, and the possibility of observations in solution is still lacking, especially for filaments characterized by a high plasticity, such as the RecA protein for homologous recombination. Here, we use a combination of SAXS measurements and integrative modeling to generate the solution structure of two known forms of the RecA nucleoprotein filament, previously characterized by electron microscopy and resolved by X-ray crystallography. The two forms differ in the cofactor bound to RecA–RecA interfaces, either ATP or ADP. Cooperative transition from one form to the other has been observed during single-molecule experiments by pulling on the filament but also in solution by modifying solvent conditions. We first compare the SAXS data against known structural information. While the crystal structure of the ATP form matches well with the SAXS data, we deduce from the SAXS profiles of the ADP-form values of the pitch (72.0 Å) and the number of monomers per turn (6.4) that differ with respect to the crystal structure (respectively, 82.7 Å and 6.0). We then monitor the transition between the two states driven by the addition of magnesium, and we show this transition occurs with 0.3 mM Mg ^2+^ ions with a high cooperativity.

## 1. Introduction

Many proteins frequently undergo self-assembly into filaments [1]. Some assemblies are disease-related, such as Tau aggregates and amyloid-β peptides [2,3]. In many cases, protein assembly is essential for their activity, as in the case of tubulin and actin. Drugs targeting the assembly and disassembly of protein filaments have been developed. Protein assembly is designed in biotechnology to perform new functions [4,5]. Despite its importance, the structural analysis of such protein assemblies is difficult because of their size and dynamics. These features make the NMR study and crystallization for X-ray crystallographic analyses difficult.

Recent technological and methodological breakthroughs have opened the way to studying large macromolecular assemblies [6,7]. Among these, filamentous oligomeric assemblies have long remained out of the scope of classical methods for atomic-level resolution, such as X-ray crystallography or NMR. Crystallizing helical assemblies either requires symmetry conditions that can be obtained only for some helical characteristics (such as an integer number of monomers per turn) [8], or necessitates system-dependent engineering procedures to restrict the extent of polymerization [9]. Following the progress in cryo-electron microscopy instrumentation and software development, the gap in structural knowledge is being filled at a large pace, and a wealth of new information on oligomeric protein association at the atomic level is now available [10,11]. These structures offer a sound basis to explore the documented plasticity of filamentous protein assemblies [12,13], and the role of this plasticity to modulate filament properties [14,15]: proteins can modify their mode of assembly in response to changes in the environment or ligand modifications such as NTP hydrolysis [16,17]. However, while metastable intermediate states can be captured via cryo-electro micrographs, these states are static. Small-angle X-ray and neutron scattering (SAXS, SANS) appear as technologies of choice to monitor the transition between metastable intermediate states in controlled conditions in terms of salt concentration, temperature, pH, and the presence of ligands or cofactors.

Helical filaments, and particularly those filaments where each helical subunit (monomer or protomer, made of one or more proteins) only binds its two next neighbors, present characteristics that need to be accounted for when addressing their structural resolution based on SAXS data. There can be numerous ways to assemble copies of a protein into helical assemblies [18], with binding energy priorities that can depend on external (pH, salt composition) or local (small molecule binding) conditions; hence, there is an intrinsic ambiguity when reconstructing protein filaments [19]. On the one hand, small variations at the protomer–protomer interface due to thermal motions [20] can produce large variations in the overall shape [18]; Egelman and collaborators reported large amplitude variations of the pitch of nucleoprotein filaments of homologous recombination in their pioneering exploration of filaments via electron microscopy [13] (for a definition of helical parameters, see Supplementary Information 1 and Figures S1 and S2). On the other hand, different protomer–protomer binding modes can lead to similar architectures in terms of pitch and number of monomers per turn [13,18]. While methods have been developed to take into account conformational ensembles of single proteins when generating SAXS data, such as [21,22,23,24], there are only a few examples in the literature where the conformational plasticity of protein filaments has been studied in solution using the SAXS methodology.

In this work, we focus on the nucleoprotein filaments formed by the polymerization of recombinase RecA on single-stranded DNA. These filaments represent an interesting test system as they are known to polymerize under at least two distinct helical forms in physiological conditions, depending on whether the protein cofactor is ATP or ADP. The form obtained with ATP is a more extended helical filament than that with ADP [13]; we will refer here to these forms as “extended” and “compressed”, respectively. Early SAXS studies on RecA filaments considered the protein as a sphere [25,26] or a cylinder [27] and compared the theoretical curves obtained at various pitch values to the experimental SAXS signal. With this aim, they took advantage of the helical symmetry of the protein filaments. More recently, an analytical formulation was developed to account for the relationship between the scattering curve parameters and parameters such as the helical pitch, the cross-sectional radius of gyration, the diameter of the helix, and the size of the protein subunits [28], opening the way to the automatic adjustment of the structural parameter to fit the scattering curve. However, approaches based on analytical formulation do not enable the exact shape of individual protein monomer to be taken into account, nor can they distinguish between different modes of filaments assembly that would produce similar helical parameters. These limitations may be bypassed using a more recent dummy atom-based method specifically developed to account for helical filaments and stacked polymers [29]. Even so, for the dummy atom method as well as the analytical method, the polymer needs to be long enough to be considered infinite, which may not be the case for many studies of biological helical filaments, including the present study, where self-assembly is a dynamic process. In our case, the structure and the dynamic behavior of the monomers are known and have been well characterized [8,9,30]; we therefore aim to build on that knowledge rather than ab initio determining the whole structure, and we also aim to concentrate on the monomer–monomer binding geometries in the observed filaments.

The “down-to-top” approach we develop here starts from sampled protein–protein interfaces to access the filament global shape, an approach that aims at complementing the panoply of tools already used for integrative modeling based on SAXS data [31]. It is appropriate for cases where the structure of the protomer is known. The approach starts by generating sets of filament models from sampled protomer–protomer binding geometries using our PTools/Heligeom tool [18,32]; the models are then used to generate ensembles of theoretical scattering curves [33] that are compared to the experimental SAXS signal. In the present case, by using ensembles of protein–protein binding geometries generated in a former study [18], we could derive relationships between peak positions and helical parameters; this enabled determining the helical parameters of the two main filament forms of the RecA protein in solution. For the extended form of the nucleoprotein filament, we find a very good agreement between the resulting solution structure and the crystal structure of short engineered one-turn filaments [9]. Conversely, the helical parameters that were obtained for the compressed form in the solution substantially deviate from the parameters of the crystal structure, which could only be crystallized with an integer number of monomers per turn [8]. We show that the filament model constructed based on the solution structure helical parameters is compatible with the binding of a single-stranded DNA, and we propose an atomic-level structural model for the compressed RecA filament with bound single-stranded DNA.

Finally, we build on the filament form characterization to monitor the magnesium dependency of the transition between the extended and compressed forms of the filament. Previous studies showed that both ATP (or analog) and Mg ^2+^ are required for the strand exchange activity of RecA [34], although RecA can form a filament with ssDNA without ${\mathrm{Mg}}^{2+}$ and ATP [8,35]. High ${\mathrm{Mg}}^{2+}$ concentrations (10 mM) are required for the optimal activity [34,36]. It is also reported that ATP alone without ${\mathrm{Mg}}^{2+}$ decreases the binding affinity of RecA to ssDNA, while ATP improves the affinity in the presence of ${\mathrm{Mg}}^{2+}$ [37]. Despite the importance of the Mg2+ ion on the RecA activities, the effects of ${\mathrm{Mg}}^{2+}$ on the structure of the RecA–ssDNA complex with ATP had not been investigated. Here, we examine the RecA–ssDNA filament structures with and without ATP and ${\mathrm{Mg}}^{2+}$.

## 2. Results

### 2.1. Estimation of the Filament Length Based on Experimental SAXS Signals

**Figure 1:**
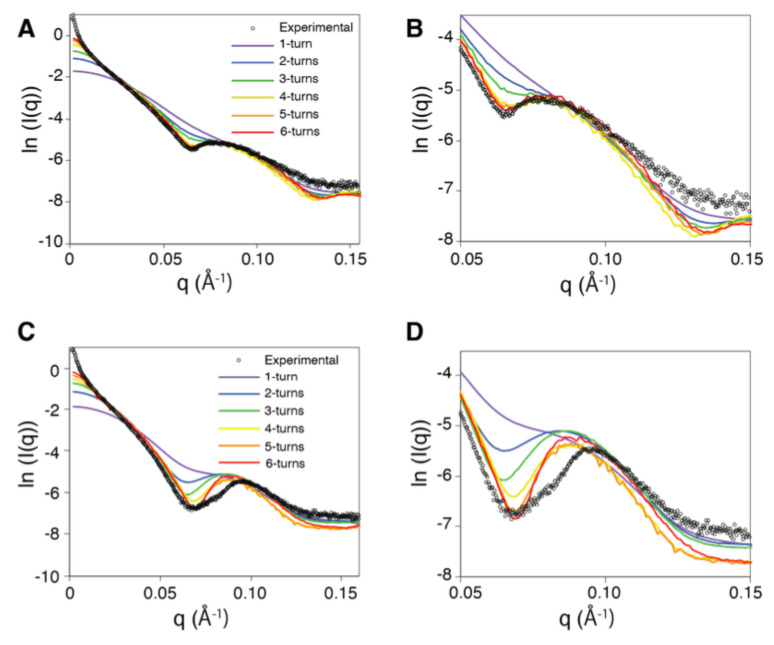
Estimating the filament length for the extended (**A**,**B**) and the compressed (**C**,**D**) forms of the RecA nucleoprotein filament. Panels (**B**,**D**) show a close-up of the curves in (**A**,**C**), restricted to *q* values greater than $0.05\;{Å}^{-1}$. The experimental SAXS signal (open black circles) is shown together with theoretical scattering curves obtained using FoxS from filament models of different lengths constructed from the crystal structures with PDB code 3CMW (**A**,**B**) and 2REB (**C**,**D**). The inserts in panels (**A**,**C**) provide the color codes associated with the different filament lengths. The insert in panel (**A**) applies to panels (**A**,**B**); the insert in (**C**) applies to panels (**C**,**D**). Intensities I(*q*) are in ${\mathrm{cm}}^{-1}$.

Former electron microscopy observations showed that at high concentrations, RecA/oligo DNA filaments can connect to each other through their extremities and form long filaments of heterogeneous sizes [27]. In agreement with these observations, the scattering intensities we observed for RecA/oligo DNA assemblies in our SAXS experiments showed a steep increase toward lower *q* values, indicative of the filament size heterogeneity (Figure 1A,C; see also Supplementary Information Figure S3). This was observed for nucleoprotein filaments both in their extended state and in their compressed state (obtained, respectively, with or without ATPγS and magnesium; see Section 3). Filament size heterogeneity makes the analysis via SAXS experiments and modeling difficult. However, attempts to proceed at low RecA concentrations led to unstable assemblies, and we were unable to obtain clear scattering profiles. We thus examined whether the well-defined broad peak situated in the vicinity of *q* = $0.1\;{Å}^{-1}$, which we will call the “first peak”, could provide information about the overall architecture of the filament, irrespective of the filament size heterogeneity. We therefore examined the effect of filament size on the SAXS profile, particularly focusing on the region with *q* values between 0.03 and $0.15\;{Å}^{-1}$, where significant information regarding the filament shape can be found [25,26,27,28,29]. The idea was to identify an average filament size that would be representative of the measured sample. For both the extended and compressed cases, we started from monomer–monomer binding geometries extracted from the crystal structures (3CMW for the ATP form and 2REB for the ADP form), and we used the Heligeom tool to generate filaments made of *x* turns, with *x* varying from 1 to 6. Figure 1 represents the theoretical scattering curves generated from these models (colored lines) using the FoxS website (https://modbase.compbio.ucsf.edu/foxs/, accessed on 13 April 2025) [33] and compared to the experimental curve (in black). For reference, given an established stoichiometry of 3 nucleotides per monomer in the extended form [9,38], an exact coverage of the 54-nucleotide DNA oligomer by the protein would produce filaments with 18 monomers, which makes approximately three helical turns. Figure 1 indicates that the observed filament size can be represented by five filament turns both for the extended and for the compressed forms (see also Supplementary Information Table S1, entries 3CMW_1 to 3CMW_6, 2REB_1 to 2REB_6). The interpretation of this number of five filament turns is not straightforward. Aggregates with large masses that dominate the lower *q*-value hump region probably interfere with the first-peak region [39]; there is presently no way of establishing the composition of the oligomeric fraction of the sample that mostly contributes to the first peak region, whether it consists of homogeneous ensembles of filaments or it displays a heterogeneous distribution of nucleoprotein filaments with different sizes, as observed in former electron microscopy studies. However, what is important here is whether the helical characteristics of the five-turn models can be related to the observed SAXS profiles and enable specifying the binding geometry within the filaments.

### 2.2. The Solution Structure of the Extended Form of the RecA Filament Matches the Crystallographic Structure

**Figure 2:**
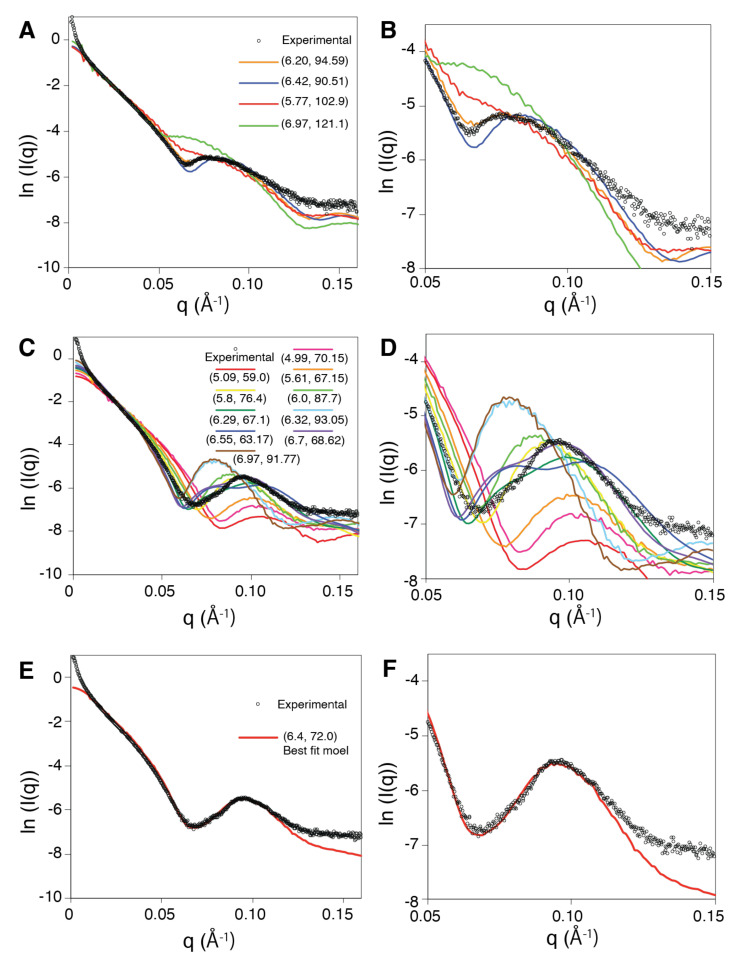
Scattering curves for (**A**,**B**) extended and (**C**–**F**) compressed forms of the RecA nucleoprotein filament. The experimental curve is represented with black circles, while curves reconstructed from models using SAXS are shown with colored lines. Color codes associated with (P, N) values are provided as inserts of panels **A** (**A**,**B**), **C** (**C**,**D**), and **E** (**E**,**F**). In panels (**B**,**D**,**F**), only the region of the curve with angle values ranging from 0.05 to $0.15\;{Å}^{-1}$ is shown, emphasizing the variability in the position of the first minimum $q_{min}$ and the first maximum $q_{max}$. Intensities I(*q*) are in ${\mathrm{cm}}^{-1}$.

We investigated more precisely the correspondence between the predicted scattering curve generated from the crystal structure 3CMW (five filament turns) and the experimental curve. To this aim, we generated filament structures presenting a large range of values for the pitch (P) and the number of monomers per turn (N) but with interfaces close to that of the crystal structure. These interfaces were retrieved from a former Monte Carlo sampling of the relative position of two monomers under the condition that at least 50% of the residue–residue contacts across the ATPase domain interface are conserved [18]. This condition ensures that the sampled interfaces can be reached from one another via thermal motions [18,20]; interfaces that differ by more than 50% of their residue-residue contacts are considered to represent alternative binding modes [18,40]. We also note that the ratio of conserved residue–residue contacts across the ATPase (core) domain interface between the crystal structures of the extended and compressed form is 0 [18]. From the structure of two bound monomers, corresponding to one given binding geometry (or one given interface), Heligeom calculates the helical (N, P) parameters of the filament that would result from uniform construction via the binding geometry. Using this tool, we selected from the sampled interfaces three binding geometries leading to diverse helical parameters, ranging from 5.7 to 7 for N and from 90.1 to 121.1 Å for P (entries E_Model_1 to E_Model_3 in Table S1, Supplementary Information 2). Figure 2A,B show the theoretical scattering curves that correspond to these geometries, together with the curve corresponding to the geometry in the crystal (N = 6.2, P = 94.6 Å) and the experimental signal. The figure shows a good correspondence between the curve obtained from the crystal structure and the experimental signal ($\chi^{2}$ = 15.8 for *q* values between 0.03 and 0.15; see Supplementary Information Table S1, entry 3CMW_5). This result supports the validity of our approach to characterize the geometry of the nucleofilament despite the filament size heterogeneity. Actually, the crystal structure of the extended form, which was obtained by covalently linking engineered proteins within very short filaments [9], can be considered to be in a relaxed form. A major sign that this conformation is relaxed is the presence of a bound oligonucleotide in the crystal. In contrast, all known crystal structures of the compressed form lack the bound DNA, even when DNA was present during the filament assembly. This may indicate that the crystal could form only at the expense of topological stress.

### 2.3. Solution Structure of the Compressed Form of the RecA Filament

**Figure 3:**
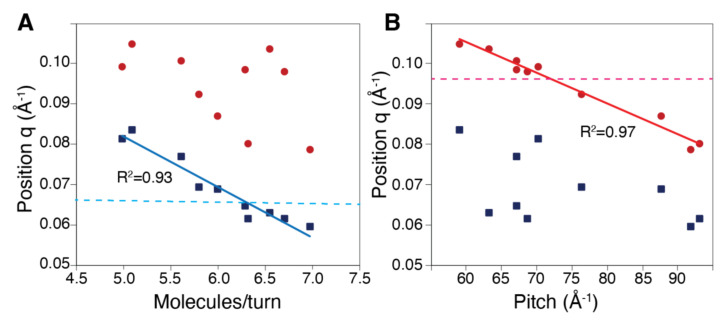
Correlations between (**A**) the number of monomers per turn and (**B**) and the pitch values and the position of $q_{min}$ (blue squares) and $q_{max}$ (red circles). The best-fitted thin lines are colored with the same color codes. Horizontal broken straight lines, blue for $q_{min}$ in the (**A**) panel and red for $q_{max}$ in the (**B**) panel indicate the experimental $q_{min}$ and $q_{max}$ values.

In contrast to the extended RecA filament form, the observed SAXS profile does not completely match the profile reconstructed from 5 turns of the crystal structure with PDB code 2REB (Figure 1C,D): while the *q* position of the first minimum in the scattering curve (which we will call $q_{min}^{exp}$) and the corresponding ln(I(*q*)) value are well fitted, the same is not true of the position and ln(I(*q*)) value of the first maximum (maximum of what we called the first peak, called $q_{max}^{exp}$). We therefore pursued investigations as described above for the extended filament form. Again, we selected interfaces close to that characterizing the crystal structure from an ensemble of Monte Carlo sampled interfaces that maintained at least 50% of the reference residue–residue contacts [18]. The selection was made in such a way as to sample a large range of (N, P) values distributed between 5.1 and 7.0 for N and 59.0 and 93.1 Å for P. We also included the model constructed from the 2REB crystal structure, characterized by a number of monomers per turn of exactly 6.0 and a pitch or 87.7 Å (entries C_Model_1 to C_Model_10 in Table S1, Supplementary Information 2). Figure 2C shows the theoretical curves corresponding to five-turn filament models constructed from the selected interfaces, and Figure 2D shows a close-up graphics for *q* values greater than 0.05. As can be noticed, none of the theoretical curves match both the ($q_{min}$, ln(I($q_{min}$)) and ($q_{max}$, ln(I($q_{max}$)) positions. However, we observed a downward shift of the theoretical $q_{min}$ values as the N values increased. In the same way, the theoretical $q_{max}$ positions seemed to decrease when increasing the pitch value. Such dependency on P of the peak position is in line with the relationship obtained from analytical approaches for infinite filaments [28]. Based on these observations, we looked for possible correlations between the positions of the $q_{min}$, $q_{max}$ values and the corresponding N and *p* values. Indeed, Figure 3 shows a very strong correlation between $q_{min}$ and N on the one hand and $q_{max}$ and P on the other hand, with the square value of the Pearson product–moment correlation coefficient $R^{2}$ exceeding 0.9 in both cases. The intersection between the interpolated best fitting straight lines for ($q_{min}$, N) and ($q_{max}$, P) and the experimental $q_{min}^{exp}$, $q_{max}^{exp}$ values (respectively 0.067 and $0.097\;{Å}^{-1}$) indicates that the solution structure is characterized by N = 6.4 monomers per turn and P = 72.0 Å.

**Figure 4:**
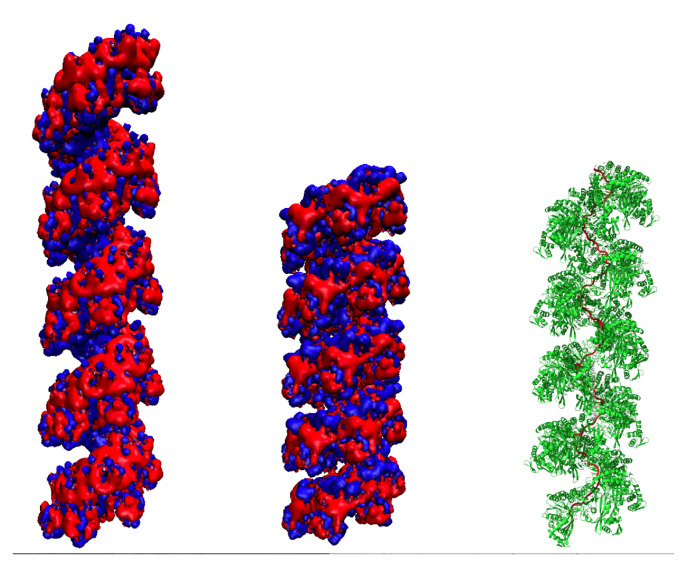
Structure and electrostatic properties of five helical turns of extended (**left**) and compressed (**middle**,**right**) forms of the RecA filament that correspond to the SAXS data. Electrostatic isosurfaces close to the protein surface, with values of −1 and 1, are represented in red and blue, respectively. Electropositive potential accumulates in the wide filament groove of the extended form, forming continuous tracks. Conversely, the compressed form presents a straighter groove, with discontinuous patches of electropositive and electronegative potential. The electrostatic potential was calculated in 0.15 mM NaCl using the APBS and PDB2PQR softwares (respectively version 3.4.1 and 3.6.1) via the webserver https://server.poissonboltzmann.org/, accessed on 14 April 2025, [44], and visualized using the Visual Molecular Dynamics (VMD) software [45], version 1.9.4. The right panel displays a cartoon structure of the compressed filament with bound DNA and ATP in the absence of magnesium.

We then generated a filament model with helical characteristics corresponding to these SAXS-derived parameters. To this aim, we used two different ways to obtain the desired binding geometry, which take advantage of the Heligeom tool to directly relate interfaces to the (N, P) helical parameters. In a first approach, we selected interfaces with the desired (N, P) values from the post-processed results of molecular dynamics simulations on a filament with mixed ATP/ADP interfaces [41]. From the two interacting monomers that define the selected interface, it was then easy to construct five-turn filaments. However, each protein structure that participated in the selected interface showed some distortion with respect to the crystal structure due to thermal moves. We therefore followed a second approach using a Heligeom-based PTools script that can adjust a helix to given helical parameters (see Section 3). In this approach, the position and orientations of one RecA protein with respect to the fixed helical axis are sampled under the condition that at least 30% of the contacts across the interface with the neighboring protein are maintained during the search. Using that script and starting from the 2REB crystal structure, we obtained a helix with exactly 6.4 monomers per turn and a 72 Å pitch, where 66% of the interface residue–residue contacts were maintained (Heligeom analysis [32]; see Figure 4, middle and right for the resulting five-turn filament). The theoretical SAXS profile calculated from five turns of the best-fitting model shows satisfying correspondence with the experimental curve, with a $\chi^{2}$ value of 13.0 for *q* values in the interval [0.03, 0.15] (Supplementary Information Table S1, entry “Best-fit model”). In order to further test the adjustment method, we applied the same script to two interacting monomers taken from another crystal structure of the compressed form of the RecA filament, with PDB code 4TWZ [42]. Interestingly, while the interfaces in the 2REB and 4TWZ structures only share 67% of their residue–residue contacts (value obtained via Heligeom calculation), the structures nicely converged upon (N, P) adjustment towards similar assembly geometries, with 90% shared residue–residue contacts. In contrast, these adjusted structures only shared 48% of their interface contacts with the assembly geometry selected from the molecular dynamics simulation. The adjustment method, where the filament monomers remain in a relaxed state, therefore appears to be more robust than the approach relying on selection from a molecular dynamics trajectory. Finally, we could build a model of the compressed filament with DNA (Figure 4 right, Supplementary Information Figure S5 and Movie S1), in which the single-stranded DNA perfectly fits in the primary binding site (site I) with a stoichiometry of 3 nucleotides per protein. That stoichiometry corresponds to one of the two possible DNA binding geometries in the compressed form that were identified in a study by Alekseev and coll. [43], with the second one presenting 5–6 nucleotides per monomer. The study showed that although the two compressed forms are interconvertible, only the form with three nucleotides per monomer can be converted to the extended form. A structural description of that nucleoprotein filament model, until now out of experimental or structural reach, can be found in Supplementary Information 4.

### 2.4. Experimental Observation of the Mg-Driven Transition Between the RecA–ATP and RecA–ADP Filament Forms

**Figure 5:**
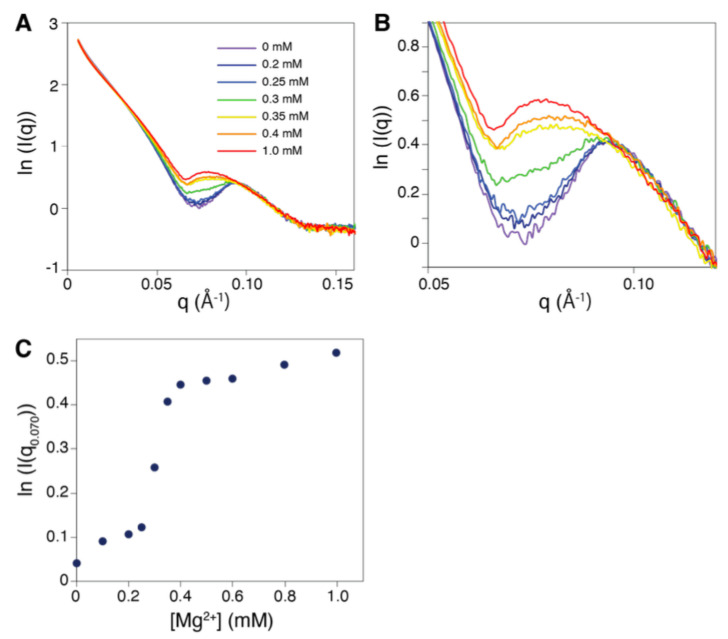
${\mathrm{Mg}}^{2+}$ titration of a RecA–ATPγS–DNA nucleofilament. (**A**) Experimental scattering curves obtained at increasing ${\mathrm{Mg}}^{2+}$ concentration; the color code is indicated as an insert; (**B**) details of the curves for *q* values between 0.05 and $0.12\;{Å}^{-1}$; (**C**) evolution of the ln(I(*q*)) values taken at *q* = $0.07\;{Å}^{-1}$ as a function of the magnesium concentration. Intensities I(*q*) are in ${\mathrm{cm}}^{-1}$.

Building on the results presented above, we then investigated the conditions for a transition between the two forms. As already stated, the extended form is usually observed when RecA proteins bind both ATP and DNA. The compressed form favors ADP binding, but it can also be obtained in the absence of cofactor and even in the absence of DNA. Te transition from the extended to the compressed form is known to occur spontaneously and cooperatively upon ATP hydrolysis, when no ATP regeneration system is present. Cooperative transition from the compressed to the extended form was observed under stretching load of bound DNA [46,47], when raising the salt concentration to high values [48,49] or by introducing non-hydrolyzable ATP analogs. Our observation of the SAXS signal under increasing magnesium concentrations (Figure 5A,B) shows that the presence of ATP or analogs by itself is insufficient to drive the transition from compressed to extended form. The signal of the filament without ${\mathrm{Mg}}^{2+}$ presents the characteristic of a compressed form ($q_{max}$ = $0.09\;{Å}^{-1}$). The signal shifts to an extended form ($q_{max}$ = $0.075\;{Å}^{-1}$) upon adding ${\mathrm{Mg}}^{2+}$. Titration of ln(I(*q*)) for *q* = $0.07\;{Å}^{-1}$, where the signal change is maximum, precisely sets the transition at 0.3 mM, in a highly cooperative way (Figure 5C), which corresponds to a magnesium ion binding to one interface over three in the filament. We note that for ${\mathrm{Mg}}^{2+}$, the concentration value that leads to the compressed-to-extended transition is hardly comparable to what is observed with ${\mathrm{Na}}^{+}$ or $K^{+}$, where the transition occurs three orders of magnitude higher (hundredths of mMol) [48,49]. This observation suggests that a precise binding of magnesium ions at the interface may be at work in the transition, while for sodium or potassium, the transition may result from a change of the overall electrostatic field.

## 3. Materials and Methods

### 3.1. Sample Preparation of RecA Protein and RecA Protein Filaments

The expression and purification method of full-length RecA from *Escherichia coli* were described previously without modifications [42,50]. The purified RecA in 50% glycerol was stored at −30 °C until used. The buffer was exchanged during dialysis for 30 mM Tris-HCl pH 7.5 including 30 mM KCl and 0.1 mM EDTA.

### 3.2. Small-Angle X-Ray Scattering (SAXS)

All the data of SAXS were collected at beamline BL40B2 at SPring-8 (Hyogo, Japan). The X-ray energy was set at 12.4 keV (wavelength: 1.0-Å) with a flux of around 3 × $10^{10}$ photons/s at the sample position, and the sample-to-detector distance was 2.2 m. The *q*-spacing, *q* = 4πsinθ/λ (where 2θ is the scattering angle and λ is the wavelength of the incident X-ray in Å), was calibrated using diffraction from silver behenate (layer spacing of 58.38 Å). The sample’s thickness for the X-ray path length was 3 mm, and the sample cell’s windows were 0.02 mm thick quartz plates (Atock Co. Ltd., Ibaraki, Japan). The 30 μL sample of 3 mg/mL (79 μM) RecA with and without 200 μM ATPγS, 0–10 mM ${\mathrm{Mg}}^{2+}$ and 237 μM single strand DNA (poly-dT, 54 bases) is placed in the cell. The 2D scattering images were collected using a PILATUS3 S 2M detector (DECTRIS, Baden-Daettwil, Switzerland) with a data acquisition time of 10 s./f for 6 frames. Preliminary data analysis and reduction were performed using ScÅtter software (v 3.1v, developed by Dr. Robert Rambo, available at https://bl1231.als.lbl.gov/scatter/, accessed on 14 April 2025) to the extent that there were no differences in the scattering curves for protein solution and solvent buffer every 10 s, respectively. The scattering curves are shown in this work for *q* values below $0.15\;{Å}^{-1}$. For higher values, the effects of thermal moves or the presence of solvent layers in the experiment do not permit comparison with theoretical curves obtained for static structures without solvent.

### 3.3. Modeling Filament Forms Based on Selected Interfaces

Filament models were constructed using Heligeom [18,32,51], a module from the PTools library [52] dedicated to the analysis and construction of helical filaments (see Supplementary Information 1; Heligeom can also be used via the webserver https://heligeom.galaxy.ibpc.fr, accessed on 14 April 2025). Heligeom can extract the helical parameters pitch (P) and number of monomers per turn (N) of a helical assembly from the structure of only two interacting protomers, which enables sampling in the (N, P) space when associated with any sampling method such as Monte Carlo, molecular dynamics or docking simulations. Other geometric parameters such as the inner and outer radius of the helical assembly are part of Heligeom output. Such parameters were used in former works for analytical formulation of scattering curves of infinite filaments [28]. In addition, Heligeom can construct filaments of any desired size based on the binding geometry at the protomer–protomer level. Finally, the PTools library contains python scripts that enable comparing interfaces in terms of $f_{NAT}$, the conserved fraction of residue–residue pairs across the interface. Interfaces are considered to be distinct when the $f_{NAT}$ value is lower than 0.5. On the contrary, interfaces with $f_{NAT}$ values greater than 0.5 can generally be deduced from each other via thermal moves and can be considered to represent the same family in terms of architecture [20,40]. We used the crystal structures with PDB codes 3CMW [9] and 2REB [8] as starting points to study the correspondence with the scattering curves of the filaments in the extended and compressed forms, respectively. Both structures are right-handed helices with close to six monomers per turn (6.2 for the 3CWM, 6.0 for 2REB) and with pitch values of 87.7 and 94.6 Å, respectively. As described earlier [18], the binding geometries between the core domains of the RecA proteins in these two filament forms are completely different and vary by more than 20 Å in terms of root mean square deviation, without any common pairs of interacting residues ($f_{NAT}$ = 0). The interface is completed by the binding of an N-terminal helix, which, in contrast, is very similar between the two forms ($f_{NAT}$ = 0.9). The binding of that N-terminal helix can adapt to different filament architectures via a flexible linker between the helix and the protein core [53] (see Supplementary Information Figure S2C). The assembly architecture is therefore completely determined by the binding geometry of the protein cores.

### 3.4. Interface Sampling and Selection

We selected interfaces with a large amplitude of (N, P) values from ensembles of sampled interfaces generated in a previous study [18]. In that former study, the ensembles had been obtained via Monte Carlo sampling: rigid body translational and rotational moves of one protein with respect to the other were randomly generated under the condition that more than half of the initial interface contact pairs were conserved and the moves were accepted or rejected based on the Metropolis criterion. In the present work, the ensembles resulting from the former study were post-processed using Heligeom to extract (N, P) values associated with each interface.

### 3.5. Interface Adjustment

Interfaces from the crystal structures of the compressed filament were adjusted to helical characteristics deduced from the analysis of SAXS curves using an in-lab python script described in the Supplementary information, Protocol S1, of reference [18]. In short, the screw transformation associated with the reference binding geometry between two protomers is calculated, which provides a starting position and orientation of the first protomer with respect to the helical axis. The second protomer is positioned with respect to the first one using the angles and translation associated with the target (P, N) values. Both protomers, represented at coarse-grained resolution [54], are then submitted to small-amplitude coordinated rotations around their centers of mass and radial translations with respect to the axis, which are accepted or rejected using a Metropolis Monte Carlo criterion after energy evaluation. Binding geometries with the best energies are retrieved.

## 4. Discussion and Conclusions

In this work, we introduce a new approach to investigate the plasticity of protein filaments in association with SAXS experiments, which explicitly take into account the variability in the binding geometry between adjacent protomers. The approach is proposed for cases where the structure of the protein building blocks is known. Structural variability of protein filaments can arise from small conformational changes of the protomer components and their binding geometries, but it can also result from the interface switching between distinct binding geometries. In both cases, the change in overall architecture resulting from these variations can vary from small to very large in a way that is often non-intuitive [18] and makes SAXS-based structural resolution difficult. Our first challenge was to deal with the heterogeneity of the observed sample, usually inherent to self-assembled protein filaments and which constitutes a hindrance to their study by SAXS methods [31,39]. We showed that even without knowing the exact composition of the observed sample in terms of the level of aggregation and the distribution and sizes of the oligomers, it is possible to identify a representative filament length that gives access to meaningful geometrical quantities. Once this representative length has been estimated, our approach relies on a preliminary exploration of possible binding geometries in association with the Heligeom tool that directly relates a binding geometry between two protomers to helical parameters such as the pitch and the number of monomers per turn. Applied to the two known forms of the RecA nucleofilament, the present work offers a way to distinguish the two forms based on the SAXS signals. Supplementary Information 3 and Figure S4 show that the distinction is possible even in the case that the filaments in the compressed and extended forms share the same helical parameters. Former attempts in the literature to fit the compressed filament structure to the scattering curve characterizing the extended form, which did not consider the binding geometries may differ when going from the compressed to the extended forms, failed to retrieve the correct structure in spite of using theoretically sound strategies [49,55]. We therefore emphasize the importance of performing extensive exploration of the favorable protomer–protomer binding geometries before attempting to fit a filament structure to an experimental scattering curve. The favorable geometries may be taken from known structures, as is the case here; they can be also identified using tools such as docking methods: for RecA, Boyer et al. [18] showed that the information about favorable interfaces is encrypted on the surface of the protein core regions.

Once possible binding geometries have been identified, investigations can be limited to exploring interfaces in the vicinity of these geometries. We could build various filamentous structures with large ranges of pitch values without changing the protomer structure but by slightly changing protomer/protomer contacts; this produced noticeable variations in the corresponding theoretical scattering curves that could be used to identify correlations between scattering values and helical parameters and extrapolate values for the solution structure. The resulting pitch value of 72 Å significantly deviates from the value of 87.7 Å measured in the early crystal structure Story et al. [8] but falls in the range of reported values obtained by EM or inferred from SAXS data [12,25,35,43,56], along with a more recent crystal structure of a RecA filament obtained without ADP or DNA [42]. The observed deviation of the number of monomers per turn when going from the crystal to the solution structure (6.0 vs. 6.4) could be expected based on the fact that crystallization of the compressed filament with exactly six monomers per turn may constrain the protomer–protomer interfaces to deviate from the most favorable (relaxed) binding geometries [9].

The RecA nucleoprotein assembly is a multicomponent complex; as such, the compatibility of each assembly component depends on the adjustment of RecA–RecA interfaces. For the extended form, it appears that the interface that makes possible the multicomponent assemblage can be stabilized only in the presence of ATP and a magnesium ion. Indeed, in the crystal structure by Chen and coll [9], a magnesium ion coordinates the cofactor ${\mathrm{ADP-AlF}}^{4+}$, a non-hydrolyzable homolog of ATP, with RecA residues. Our results indicate that the presence of this magnesium ion is necessary to stabilize that interface. They also strongly suggest that the stabilization of only one interface over three is sufficient to cooperatively drive the whole filament structure to an extended form, with homogeneous RecA–RecA binding geometries.

Constructing the filament with a correct interface is a key issue when inferring the structure from the SAXS signal. The binding geometry will determine which amino acids are accessible to the solvent or to binding a partner molecule, how the residues are distributed along the filament interior, or the width of the filament groove [18]. In the case of the RecA oligomer, the topology of the helical groove resulting from the assembly in the extended form permits binding the LexA protein and inducing its self-cleavage; conversely, the RecX protein that exerts control of the homologous recombination process only binds in the groove of the compressed form [57]. In the present model (Figure 4 right and Supplementary Information 4), the adjustment of consecutive monomers based on the SAXS-defined interface enables the formation of a deep continuous track situated between the RecA loops L1 and L2, where a DNA strand perfectly fits. This track is mostly conserved between the compressed and the extended form, which may explain the possible interconversion between the two forms in spite of a large amplitude interface change. The function is therefore tightly associated with the monomer–monomer binding mode. We emphasize the need to pay special attention to the mode of associating protomers in a helical filament in addition to recovering the global shape when modeling solution structures of filamentous assemblies based on SAXS data.

## Data Availability

All models and fitting data described in this article have been deposited in Zenodo and can be freely accessed at https://doi.org/10.5281/zenodo.14930760.

## Supplementary Materials

The following supporting information can be downloaded at https://www.mdpi.com/article/10.3390/molecules30081793/s1, Figure S1: Definition of a screw transformation; Figure S2: Helical characterization and plasticity of regular filaments; Figure S3: SAXS profile of the compressed and extended forms of the RecA nucleoprotein filament; Figure S4: Theoretical SAXS profiles of model filaments in the compressed and extended forms that present similar (N, P) helical parameters; Figure S5: Details of site I and site II in the solution structure of the compressed filament model; Table S1: Helical characterization of the model filaments used in this study and comparison of the theoretical SAXS signals generated by FoxS to experimental SAXS profiles; Table S2: Helical parameters of selected filaments with similar (N,P) parameters, either in the Compressed or in the Extended form; Movie S1: Rotating view of the compressed form of ssDNA-RecA-ATP nucleoprotein filament. Refs. [58,59] are cited in Supplementary Materials.
