# Supplementary material for: Plasticity and Co-Factor-Dependent Structural Changes in the RecA Nucleoprotein Filament Studied by Small-Angle X-Ray Scattering (SAXS) Measurements and Molecular Modeling
Source: Molecules. 2025 Apr 16;30(8):1793. doi: 10.3390/molecules30081793 (PMC12029565; doi:10.3390/molecules30081793)
Supplement: Supplementary file 1 [file molecules-30-01793-s001.zip › molecules-3482870-supplementary.pdf]

Plasticity and co-factor dependent structural changes of the RecA nucleoprotein filament studied by SAXS measurements and molecular modeling

S. Inaba-Inoue, A. Sabei, A-E Molza, T. Mikawa, H Sekiguchi, C. Prévost and M. Takahashi

Supplementary information 1 - Screw transformations and relationship between interface and helical architecture

Supplementary information 2 - Comparison of the theoretical and experimental SAXS signals of the nucleoprotein filaments described in the main text (Figures 1-3)

Supplementary information 3 - Filament plasticity and SAXS profiles

Supplementary information 4 - Model of the compressed form of ssDNA-RecA-ATP nucleoprotein filament

Table S1- Helical characterization of the model filaments used in this study and comparison of the theoretical SAXS signals generated by FoxS to experimental SAXS profiles.

Table S2 - Helical parameters of selected filaments with similar (N,P) parameters, either in the Compressed or in the Extended form

Figure S1 - Definition of a screw transformation

Figure S2 - Helical characterization and plasticity of regular filaments

Figure S3 - SAXS profile of the compressed and extended forms of the RecA nucleoprotein filament

Figure S4 - Theoretical SAXS profiles of model filaments in the compressed and extended forms that present similar (N, P) helical parameters

Figure S5 - Details of site I and site II in the solution structure of the compressed filament model.

Movie S1 - Rotating view of the compressed form of ssDNA-RecA-ATP nucleoprotein filament

Supplementary information 1 - Screw transformations and relationship between interface and helical architecture

Regular self-assembly of rigid objects leads to helical, cyclic or linear architectures via screw transformations. A screw transformation is characterized by a translation along an axis and a rotation around that axis (Figure S1). If the translation is null, the resulting assembly is a ring; if the rotation is null, then the assembly is linear. In any other case, the assembly is helical.

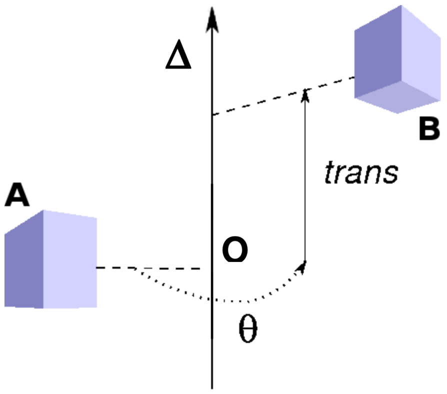

Figure S1 *Definition of a screw transformation (taken from Boyer 2015, supplementary information scheme SI-1)*

Correspondence between the screw parameters (*trans*, θ) **and the helical parameters** (*N, P*). The screw assembly can be characterized by quantities such as the pitch (*P*), the number of subunits per turn (*N*), and the screw direction (*dir*, either right-handed or left-handed) (Figure S2 A) which can be accessed experimentally. The pitch for example can be directly measured on electron micrographs or via atomic force microscopy. These geometrical parameters can be directly deduced from the screw parameters.

$$N = 360°/\theta$$

$$P = N \times trans$$

$$dir = \begin{cases} R \text{ } if \text{ } (\theta \times trans) > 0 \\ L \text{ } if \text{ } (\theta \times trans) < 0 \end{cases}$$

We note that the pair of geometrical descriptors (N, P) does not univocally determine the transformation that goes from one protomer to its neighbor. Additional parameters are the distance of the center of mass to the helix axis, which is related to the helix diameter and for non-cylindrical protomers, which is the general case, the orientation of the protomer with respect to the axis (Figure S2 B,C). Influence of these extra parameters are discussed in Supplementary information 2 below.

The Heligeom software. From the structure of two consecutive assembly subunits provided as input, Heligeom computes the screw parameters trans, θ, coordinates of the axis origin, axis unit vector. This provides a complete description of the screw transformation that relates the two subunits and enables the construction of regular helical assemblies of any length based on the provided geometry. Helical parameters (N, P) together with the inner and outer helix radii are also provided. Heligeom analyses and construction tools can be performed via the webserver https://heligeom.galaxy.ibpc.fr (Santuz 2025)

Supplementary information 2 - Comparison of the theoretical and experimental SAXS signals of the nucleoprotein filaments

PDB file name	number of turns	number of Subunits per turn	pitch (Å)	internal radius (Å)	external radius (Å)	Chi^2 ($Å^{-1}$) all range	Chi^2 [0.03-0.15A] ($Å^{-1}$)
**EXTENDED**							
3CMW_1	1	6.20	94.59	6.2	58.9	6307.2	442.3
3CMW_2	2					2358.8	99.0
3CMW_3	3					1185.4	50.4
3CMW_4	4					574.3	18.1
**3CMW_5**	**5**					412.0	**15.8**
3CMW_6	6					291.1	16.6
E_Model_1	5	6.42	90.51	8.0	60.1	344.4	16.0
E_Model_2	5	5.77	102.90	7.5	53.5	810.4	112.2
E_Model_3	5	6.97	121.06	8.5	60.2	698.9	369.7
**COMPRESSED**							
2REB_1	1	6.00	82.70	4.9	62.9	8373.7	856.7
2REB_2	2					3197.3	208.6
2REB_3	3					1709.6	142.7
2REB_4	4					1274.0	106.4
2REB_5	5					919.1	91.3
2REB_6	6					785.2	81.0
C_Model_1	5	4.99	70.15	4.7	53.4	2972.8	626.91
C_Model_2	5	5.09	59.00	3.6	54.0	2926.7	596.6
C_Model_3	5	5.61	67.15	3.8	60.3	1589.0	247.0
C_Model_4	5	5.80	76.40	3.4	63.8	779.2	28.6
C_Model_5	5	6.00	87.70	4.9	62.9	919.1	91.3
C_Model_6	5	6.29	67.10	8.4	68.7	752.4	31.7
C_Model_7	5	6.32	93.05	5.1	66.2	1077.6	710.1
C_Model_8	5	6.55	63.17	8.2	70.3	782.0	102.9
C_Model_9	5	6.70	68.62	8.0	70.4	657.6	107.9
C_Model_10	5	6.97	91.77	8.4	68.7	1206.6	1161.0
**Best-fit model**	**5**	**6.40**	**72.00**	**6.3**	**65.0**	252.0	**13.0**

Table S1 - *Helical characterization of the model filaments used in this study and comparison of the theoretical SAXS signals generated by FoxS (Schneidman-Duhovny 2016) to experimental SAXS profiles (see main text).*

Supplementary information 3 - Filament plasticity and SAXS profile

Physico-chemical properties of the filament, such as the spatial distribution of masses, the electrostatic field around the filament, the steric accessibility to the filament groove, the internal or external diameter of the filament are determined by both the helical parameters and the way proteins are oriented with respect to the filament axis (Figure S2). For a regular assembly, these properties directly derive from the binding geometry between consecutive protomers, or in other words from the binding interface. In this section, we explore whether SAXS analysis is able to distinguish between two different binding geometries that would lead to similar helical architectures in terms of pitch and number of monomers per turn.

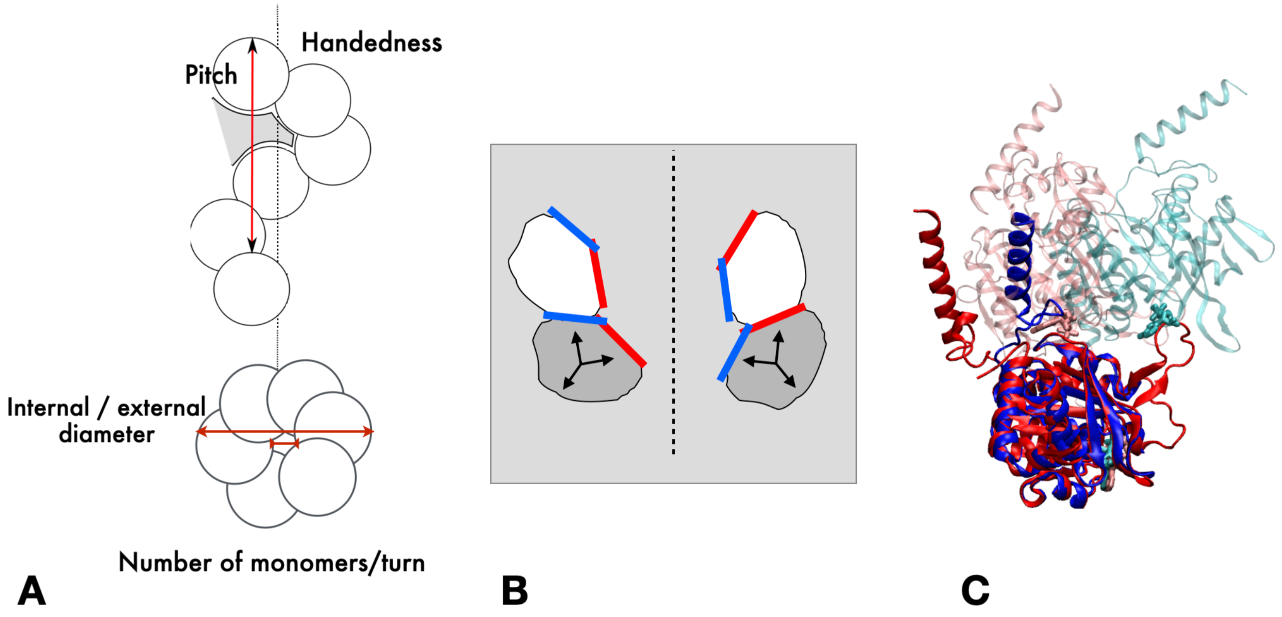

Figure S2 *Helical characterization and plasticity of regular filaments. (A) Helical parameters and geometrical characterization; the grey shaded region represents the filament groove, a region that is accessible to ligand binding and whose topology is defined by the filament geometry. (B) Tho different modes of association for a given monomer, either via the interface represented by a red line or by that represented by a blue line; within the corresponding filaments aligned on the Z axis (broken line), the monomers present different orientations with respect to the axis. (C) The two main association geometries of RecA monomers (respectively red/pink and blue/cyan for the geometries leading to extended or compressed geometries); the ATP-binding cores of the bottom monomers are superimposed, showing the relative displacement of the upper monomer with respect to their superposed binding partners, with an amplitude of 20 Å.*

Comparison between the SAXS signal characterizing extended and compressed filament forms of the RecA nucleoprotein filament. Figure S3 shows a superposition of the scattering curves obtained for the compressed (black) and extended (red) forms of the filament.

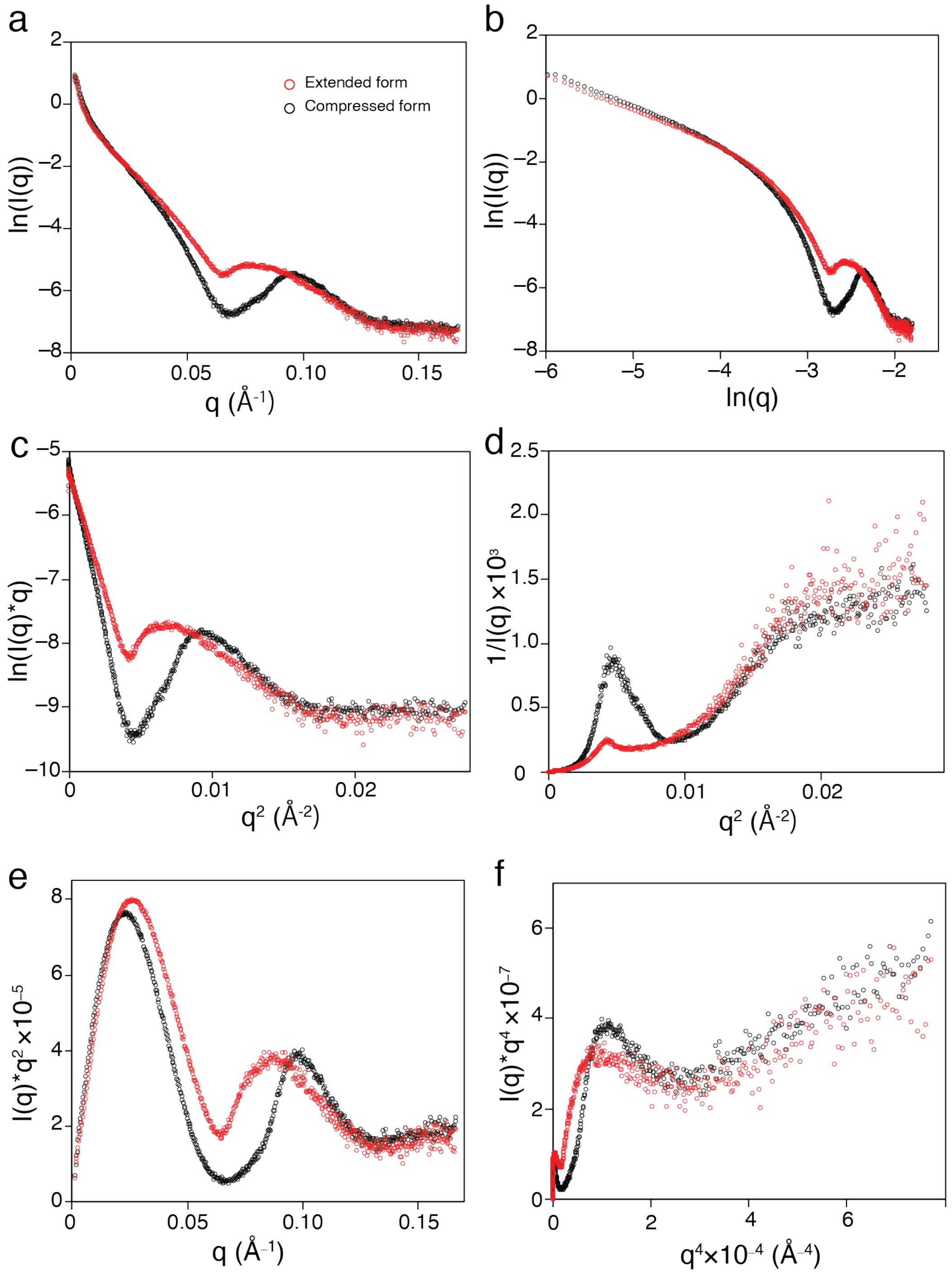

Figure S3 - *The comparison of experimental scattering curves for extended (red dotted) and compressed (black dotted) form of RecA filaments. (a) Intensity plots, (b) Double logarithm plots, (c) Guinier Rod plots, (d) Debye-Bueche plots, (e) Kratky plots, and (f) Porod-Debye plots. I(q) is in* cm^{-1}

Generation of filament models showing identical or quasi-identical (N, P) parameters. The plots shown in Figure S3 correspond to filaments where both the (N, P) helical parameters and the association geometry between successive RecA monomers vary. In order to separate out the two factors, we constructed filament geometries that share identical helical characteristics while presenting either a “compressed-like” or an “extended-like” association geometry. Identification of these geometries relied on the post-processing analysis of a molecular dynamics trajectory of a structurally composite RecA filament, that was performed in former work (Boyer 2019). In that model, designed to explore the potential effect of ATP hydrolysis in the filament, the overall structure had been assembled according to the extended geometry except for the central interface, that was taken from the compressed form. During the molecular dynamics simulation, each interface in the filament sampled geometries close to the starting interface without escaping from the potential energy well associated to that starting interface, as testified by the values of the fraction of common contact pairs (f_{NAT}) not decreasing below 0.6 (Boyer 2019, supplementary information).

In the present work, we computed (N, P) values characterizing the interface state at each step of the trajectory and for each interface. Interestingly, we found several examples of “compressed-like” and “extended-like” interfaces that presented similar (N, P) values (Table S2). This finding is in agreement with electron microscopy observations from the Egelman group of an overlap between pitch values measured in ensembles of stretched filaments or ensembles of compressed filaments (Yu 1992). In addition here, we find that not only the pitch but also the number of monomers per turn can be common to filaments that belong to two different geometrical families in terms of binding geometries, either “extended-like” or “compressed-like”. The range where the overlap is observed concerns pitch values between 73 and 91 Å and N values between 5.9 and 6.2 (Table S2). We constructed 5-turn filaments based on those selected geometries, listed in Table S2 and indexed by letters going from “a” to “h". Results displayed in Figure S4 show differences both in intensity and in peak positions.

Model	Compressed		Extended	
	number of monomers per turn	pitch (Å)	number of monomers per turn	pitch (Å)
a	6.20	90.93	6.20	90.92
b	6.16	88.63	6.16	88.64
c	5.97	88.50	5.98	88.51
d	6.00	85.91	6.01	85.91
e	6.16	84.95	6.14	84.94
f	5.83	84.06	5.84	84.05
g	6.26	83.43	6.26	83.46
h	6.02	81.53	6.02	81.54
i	5.82	81.20	5.80	81.20
j	5.82	79.30	5.82	79.29
k	5.99	77.01	6.01	76.99
l	5.90	73.89	5.89	73.90

Table S2 - *Helical parameters of selected filaments with similar (N,P) parameters, either in the Compressed or in the Extended form*

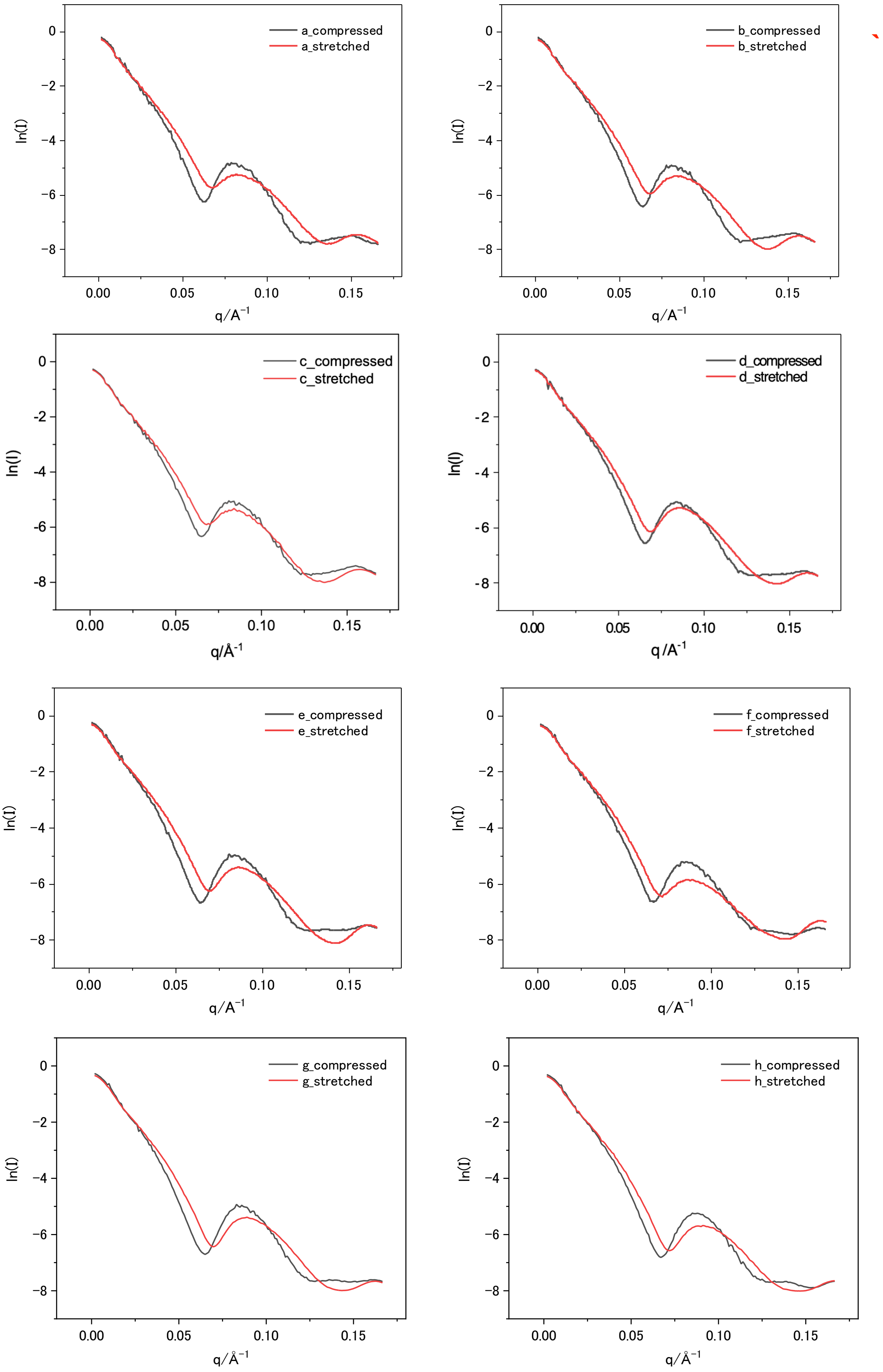
a_compressed
a_stretched
b_compressed
b_stretched
c_compressed
c_stretched
d_compressed
d_stretched
e_compressed
e_stretched
f_compressed
f_stretched
g_compressed
g_stretched
h_compressed
h_stretched
ln(I)
q/A^{-1}
q/Å^{-1}
0
−2
−4
−6
−8
0.00
0.05
0.10
0.15

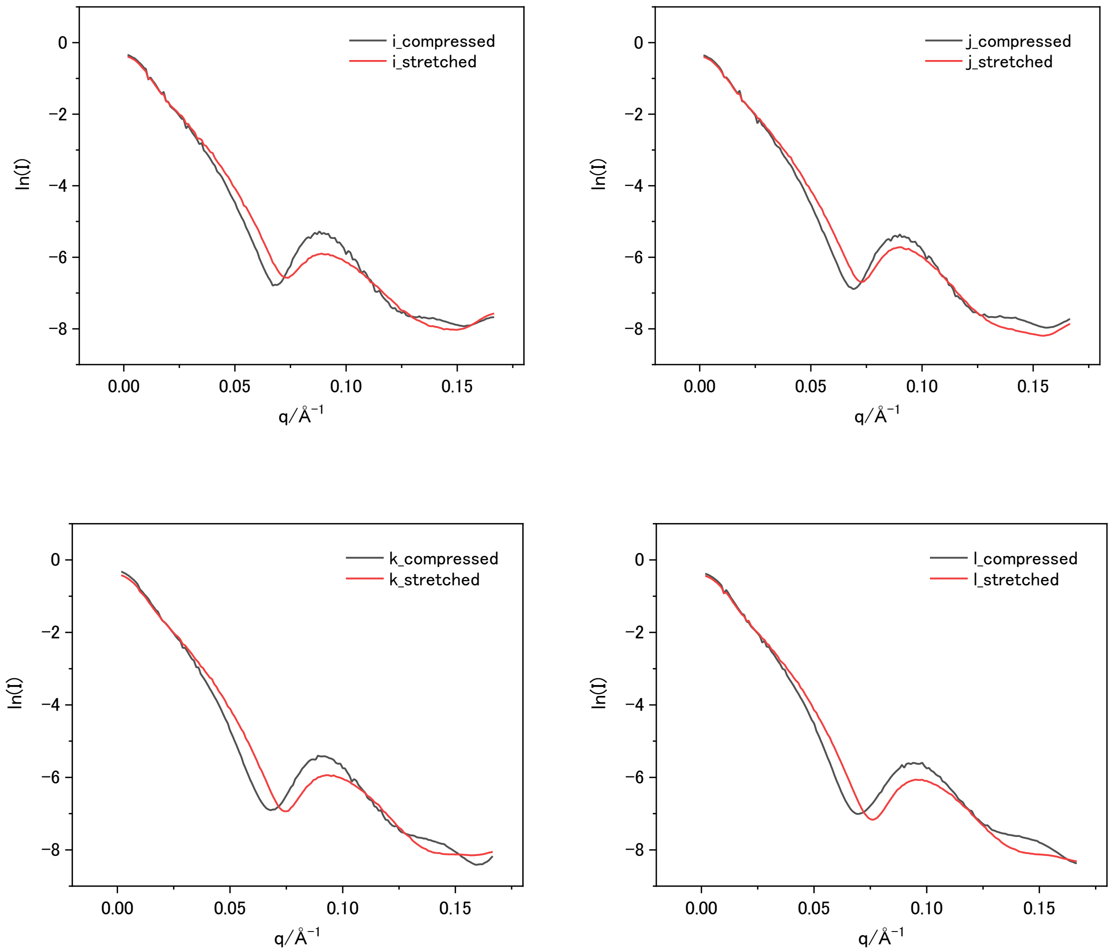

Fitting analysis

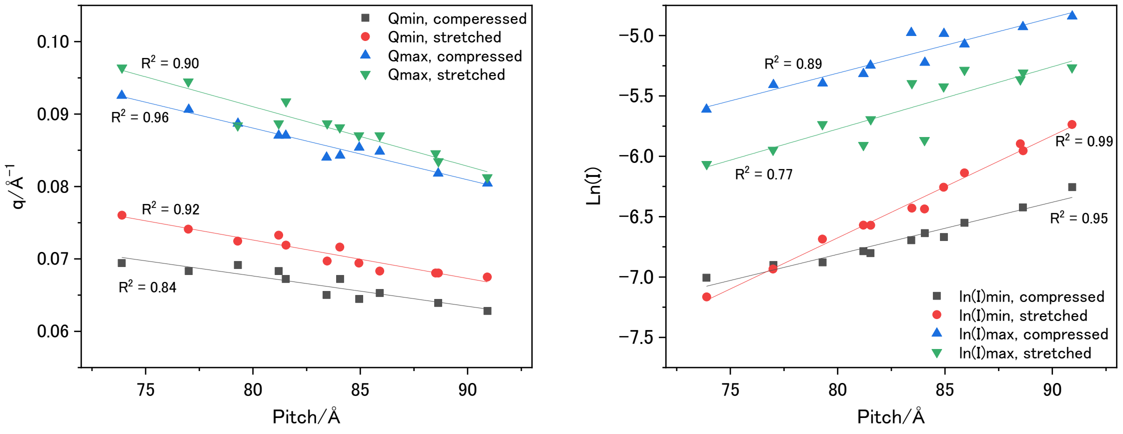

Figure

Figure S4 - *Theoretical SAXS profiles of 5-turn models a to l of compressed or extended filament presenting similar (N, P) values; the lower panel shows the correlations between q_{min} and q_{max} and pitch values (left), between minimum and maximum intensities ln(I) and pitch values (right). Interestingly, although the correlations are strong, the slopes differ whether the filaments are in compressed or extended forms; the effect is more pronounced for the minimum intensities.*

Supplementary information 4 - Model of the compressed form of ssDNA-RecA nucleoprotein filament

The approach described in this work identified the binding geometry within the compressed form of the RecA nucleoprotein filament in solution but did not account for the bound DNA. Indeed, the contribution of that oligonucleotide, bound in the interior of the filament groove, to the SAXS profile is at most very low. However, one main condition to validate the proposed structure is its compatibility with the binding of a DNA single strand. We therefore constructed a model for the compressed form of the nucleoprotein filament bound to RecA, with ATP as a cofactor (in the main article, we confirmed that ATP alone cannot drive the transition to an extended form in the absence of magnesium). In this section, we describe the modeling process, together with interesting structural characteristics observed in the final model of the compressed filament form bound to DNA.

Modeling the compressed form ssDNA-RecA-ATP nucleoprotein filament. We started from the structure of two bound RecA proteins (called A', B') in the SAXS-determined binding geometry of the compressed form (see main article). Since as we showed the compressed form can easily transition to the extended form, our guess was that the strong binding interactions of the oligonucleotide in site I in the extended form could be conserved at least in part in the compressed form. We therefore superposed to protein A' a monomer A taken from the extended filament form (pdb ID 3CWM), and we applied the same transformation (from monomer A to monomer A') to the triplet region of the oligonucleotide bound to monomer A in site I and the bound ATP molecule, giving way to subunit A (monomer A + DNA triplet + ATP) interacting with protein B' according to the A'-B' geometry. Then the dimer composed of subunit A and monomer B' was submitted to the Heligeom webserver https://heligeom.galaxy.ibpc.fr (Santuz, 2025) to construct a 3-turn filament formed by 18 repetitions of subunit A according to the A'-B' binding geometry. Although the DNA resulting from the brute construction showed discontinuous backbone, it appeared that the backbone extremities were very close and could be covalently linked with only minor adjustments. This required renumbering the nucleotides, renaming the chain fragments (chain S), and creating a topology file using the psfgen module of VMD (Humphrey, 1995). A simple energy minimization with namd 3.0.1 (Phillips, 2005), under position restraints on protein Cα atoms and on the central P atom of each triplet, produced a structure with relaxed DNA geometry. This structure was then submitted to a short molecular dynamics simulation with namd 3.0.1 to verify its stability.

Description of the solution structure of the compressed ssDNA-RecA-ATP nucleoprotein filament. The model of the compressed form of the RecA nucleoprotein filament, compatible with SAXS data obtained in solution, is represented in the main article Figure 4 (right) and in Movie S1, with details in Figure S5. We first observe that the binding site I, situated between loops L1 and L2 that line the filament interior, is well conserved between the extended and the compressed forms. This was expected since site I is also present in the crystal structure of the compressed form (pdbid 2REB), however the interface shift between the two forms might have introduced discontinuities in that site, which is not the case. To the contrary, site I appears here as a very well defined deep track between ridges made of continuous loops L2 on the one side (inner ridge) and continuous loops L1 on the other (outer ridge; respectively colored in red and yellow in Movie S1),

which easily accommodates the oligonucleotide. Loops L1 and L2 present a strong network of connections that contribute to defining the site topology (Figure S5A).

Structure of the DNA bound to the RecA compressed filament form - The first observation is that in addition to conserving the same location in site I than in the extended form, the bound oligonucleotide conserves the same stoichiometry of 3 nucleotides per RecA protein. However, contrarily to the extended form the bases are now evenly distributed, with no intercalation site between groups of 3 bases as found in the extended form and no intercalated protein residue — Ile199, that intercalates between groups of 3 bases in the extended form, remains close to the bases (Figure S5A). Indeed, the rise between bases amounts to 3.9 Å, much closer to the 3.6 Å value observed in B-DNA than to the average 5.1 Å value in the extended RecA-bound form. This base separation does not enable any residue intercalation. However, the 10% increase in rise with respect to perfectly stacked bases provides some mobility to the bases, as observed during a short molecular dynamics simulation where the oligonucleotide remained securely inserted in the site I track. We also note that the DNA in site I of the compressed form is further away from the axis than in the extended form (highest backbone distance of 16 Å *vs* 11.5 Å, shortest base distance of 6.2 Å *vs* 0.3 Å).

The rise obtained in site I also excludes the possibility of a higher stoichiometry for the DNA in that site. In an alternative geometry identified by Alekseev and coll. (Alekseev 2022) for DNA binding to the compressed form, the stoichiometry is 5-6 nucleotides per monomer. That stoichiometry can be obtained only if the nucleotide goes to site II, which is situated on the other side of loops L2 with respect to the L1 loops and is laterally displaced from the filament axis (Figure S5B). The lateral displacement of site II residues 226, 227, 243, 245 is 24 Å for the compressed form *vs* 15 Å for the extended form. Therefore, the pathway where the backbone can bind in site II (dotted line in Figure S5B) is longer than the equivalent pathway in site I, which may account for the binding of 5 nucleotides in that site. Such configuration may explain why the compressed form with the 3 nucleotide stoichiometry can easily interconvert with the extended form, while conserving the oligonucleotide in site I, although the form with 5-6 nucleotides cannot.

Cofactor location in the RecA compressed filament form - Modifying the binding geometry between RecA proteins when going from the extended to the compressed form does not modify the protein site where ADP or ATP nucleotides bind but modifies the location of that binding site in the global filament superstructure. Here, we find that although the cofactor location is shifted away from the filament axis by about 4 Å, the binding pocket opens to a solvent-accessible space in the filament central groove, which may enable cofactor exchange to take place as already described (Story 1992). Unexpectedly, we also observe that in the filament, each cofactor is located between two consecutive basic spots that characterize DNA binding site II (namely Arg226, Arg227, Arg243, Lys245, Figure S5B). We had already noticed that site II in the compressed filament is poorly defined in a steric point of view and that even in the absence of DNA and cofactors, the electrostatic potential in that site presents discontinuities (Figure 4 middle panel, main article). The location of the cofactors in a region close to the site II basic groups may increase that discontinuity.

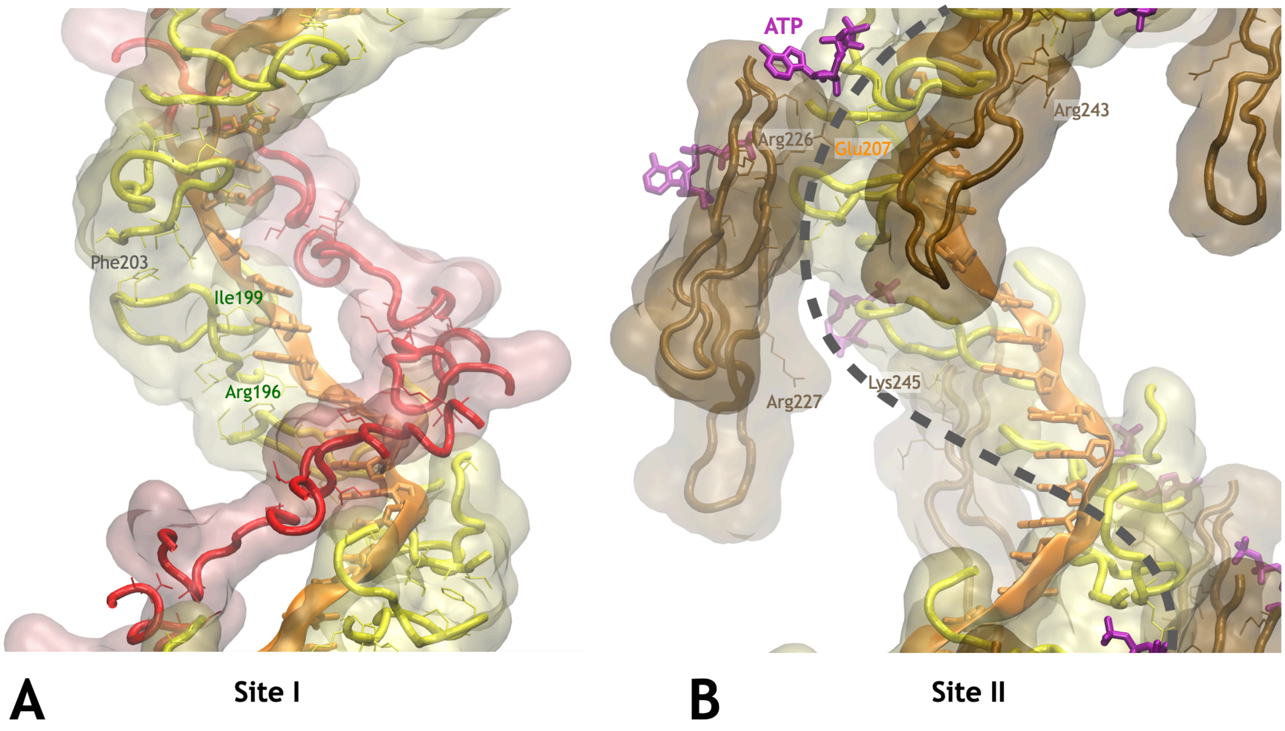

Figure S5 - *Details of site I and site II in the solution structure of the compressed filament model. (A) Site I is a deep track lined by ridges made of consecutive loops L1 (red, 156-170) on the one side and consecutive loops L2 (yellow, 194-212) on the other side. Consecutive L1 loops contact each other (residues 159, 160, 164, 169 represented in licorice, red) and consecutive L2 loops stack on top of each other, notably through Phe203, a residue that is involved in strand exchange in the extended form (Yang 2015). The DNA strand (orange) mainly contacts loop L2, with Arg196 interacting with the backbone phosphates and Ile199 making hydrophobic contacts with the bases. (B) Site II, represented by a broken line, lies between L2 loops (yellow) and hairpins (ochre, 223-245) that anchor the L2 loops via salt bridges between Arg226 and Glu207. The hairpins contain patches of basic residues Arg226, Arg227, Arg243 and Lys245 that can bind a DNA strand. The ATP cofactor is represented in licorice and colored in purple. In panels A and B, the motifs (loops, hairpins) are represented by their fold (tube), the volume they occupy (surface, transparent) and labelled residues in licorice.*

References

Schneidman-Duhovny, D.; Hammel, M.; Tainer, J.A.; Sali, A. FoXS, FoXSDock and MultiFoXS: Single-state and multi-state structural modeling of proteins and their complexes based on SAXS profiles. *Nucleic Acids Res* **2016**, 44, W424–9.

Boyer, B.; Danilowicz, C.; Prentiss, M.; Prévost, C. Weaving DNA strands: structural insight on ATP hydrolysis in RecA-induced homologous recombination. *Nucleic Acids Res* **2019**, 47, 7798–7808.

Yu, X.; Egelman, E.H. Structural data suggest that the active and inactive forms of the RecA filament are not simply interconvertible. *J Mol Biol* **1992**, 227, 334–46.

Santuz, H.; Laurent, B.; Robert, C.H.; Prévost, C. Heligeom: A web resource to generate, analyze, and visualize filament architectures based on pairwise association geometries of biological macromolecules. *J Mol Biol* **2025**, 169019.

Humphrey, W.; Dalke, A.; Schulten, K. VMD: visual molecular dynamics.J Mol Graph1996,14, 33–8, 27–8.

Phillips, J. C.; Braun, R.; Wang, W.; Gumbart, J.; Tajkhorshid, E. ;Villa, E.; Chipot, C.; Skeel, R. D.; Kalé, L.; Schulten, K.. Scalable molecular dynamics with NAMD. *J Comput Chem*, **2005**, 26, 1781–1802.

Alekseev, A.; Morozova, N.; Vedyaykin, A.; Yakimov, A.; Khodorkovskii, M.; Pobegalov, G. Single-molecule characterization of compressed RecA nucleoprotein filaments. *Biochem Biophys Res Commun* **2022**, 614, 29–33.

Yang, D.; Boyer, B.; Prévost, C.; Danilowicz, C.; Prentiss, M. Integrating multi-scale data on homologous recombination into a new recognition mechanism based on simulations of the reca-ssdna/dsdna structure *Nucleic Acids Res* **2015**, 43, 10251-63.

Story, R.M.; Weber, I.T.; Steitz, T.A. The structure of the E. coli recA protein monomer and polymer. *Nature* **1992**, *355*, 318–325
